# The Genetics of Major Depression

**DOI:** 10.1016/j.neuron.2014.01.027

**Published:** 2014-02-05

**Authors:** Jonathan Flint, Kenneth S. Kendler

**Affiliations:** 1 Wellcome Trust Centre for Human Genetics, Roosevelt Drive, Oxford, OX3 7BN; 2 Virginia Commonwealth University, Department of Psychiatry, Virginia Institute for Psychiatric and Behavioral Genetics, Richmond, VA 23298-0126, USA

## Abstract

Major depression is the commonest psychiatric disorder and in the U.S. has the greatest impact of all biomedical diseases on disability. Here we review evidence of the genetic contribution to disease susceptibility and the current state of molecular approaches. Genome-wide association and linkage results provide constraints on the allele frequencies and effect sizes of susceptibility loci, which we use to interpret the voluminous candidate gene literature. We consider evidence for the genetic heterogeneity of the disorder and the likelihood that subtypes exist that represent more genetically homogenous conditions than have hitherto been analyzed.

## Teaser

Genetic analysis of major depression lags behind that of other disease. Genome-wide association and linkage results provide constraints on major depression’s genetic architecture, indicating that candidate gene studies are underpowered. The disorder is heterogeneous; subtypes likely exist that represent more homogenous conditions suitable for genetic dissection.

## Main Text

### Introduction

A lot is being asked of the genetic analysis of major depression (MD): to find the biological underpinnings of one of the commonest psychiatric illnesses and one of the world’s leading causes of morbidity. While lifetime prevalence estimates vary, from 3% in Japan to 16.9% in the U.S., in all countries the disorder is common, with a frequency typically varying from 8% to 12% (Demyttenaere et al., 2004, Kessler et al., 2003). In the U.S., MD has the greatest impact of all biomedical diseases on disability; in Europe, it is the third leading cause of disability (Alonso et al., 2004b, Nierenberg et al., 2001, Penninx et al., 2001, Ustün et al., 2004).

Despite its prevalence and MD’s enormous burden on our health care systems (Scott et al., 2003), our treatments are almost entirely symptomatic. There is even dispute about the value of medication (Khin et al., 2011, Kirsch et al., 2008, Turner et al., 2008, Vöhringer and Ghaemi, 2011) and psychological therapies (Cuijpers et al., 2008, Cuijpers et al., 2010, Cuijpers et al., 2011). Genetic analysis, by identifying risk variants and thereby increasing our understanding of how MD arises, could lead to improved prevention and the development of new and more effective therapies.

Although genetic analysis has identified risk loci for many other common medical diseases (Hindorff et al., 2009), success has yet to visit MD. In this Review, we consider what has so far been learnt, consider reasons for the difficulties encountered, and propose how these might be overcome. We start by reviewing evidence from the genetic epidemiology literature relevant to the genetic basis of MD. We then consider what genome-wide association studies (GWASs) have told us. The GWAS results are particularly important for interpreting the large, forbidding literature on candidate gene studies, which we review next. In addition, GWAS findings inform us about the extent to which rare but more highly penetrant genetic variants might contribute to liability to MD. We finally examine whether there exist forms of MD that might be more genetically homogeneous and consider how these might be identified.

### Genetic Epidemiology

Studies showing that MD aggregates within families date back to the early decades of the 20^th^ century (reviewed in Tsuang and Faraone, 1990). Meta-analysis of the highest-quality family studies produced an estimated odds ratio for increased risk for MD in first-degree relatives of MD probands of 2.84 (Sullivan et al., 2000). Surprisingly, no high-quality adoption study of MD has been performed, so our evidence of the role of genetic factors in its etiology comes solely from twin studies. While the first of these also date to early in the 20^th^ century, only six high-quality studies were identified in the Review completed in 2000 (Sullivan et al., 2000). Meta-analysis estimated heritability for MD to be 37% (95% confidence intervals 31–42). There was no evidence from these studies that shared environmental factors contributed meaningfully to the familial aggregation for MD. One particularly large-sample twin study of MD estimated the heritability of MD at 38% (Kendler et al., 2006).

Epidemiological studies of MD have consistently shown a higher prevalence rate for women (Weissman et al., 1993, Weissman et al., 1996). Therefore, twin researchers have been interested in asking whether the heritability of MD differs across sexes and, more interestingly, whether the same genetic factors impact on risk for MD in men and women. The two major studies that have addressed this question found reassuringly similar answers (Kendler et al., 2001, Kendler et al., 2006). In both studies, MD was appreciably more heritable in women than in men (40% versus 30% and 42 versus 29%, respectively) and clear evidence was found for sex-specific genetic effects with genetic correlations estimated at +0.55 and +0.63. A substantial proportion of genetic risk factors for MD appeared to be shared in men and women. However, these results also predict that when the individual genetic variants that impact on risk for MD are definitively characterized, an appreciable proportion of them will be relatively sex specific in their effect.

### Genome-wide Association Studies

Table 1 summarizes the nine published genome-wide association studies for MD. GWASs are typically carried out in two stages: a discovery phase, in which the entire genome is screened, and a replication phase, in which a subset of SNPs are tested in an independent cohort. Some studies report the replication and discovery results separately; others combine the p values of all studies (including the discovery sample) in a meta-analysis. Information on sample sizes for the two phases is shown in Table 1.

**Table 1:** Summary of Genome-wide Association Studies of Major Depression

Sample Origin	Sample	Cases	Controls	SNPs	Phenotype	Marker	OR	p Value	Position
**Lewis et al., 2010**									

UK	Discovery	1,636	1,594	471,747	RMD	rs9416742	0.719	1.30 × 10^−7^	chr10:60542444
UK	Meta	1,418	1,918	–	–	rs606149	1.248	2.57 × 10^−6^	chr1:193921298

**Muglia et al., 2010**									

Europe	Meta (two samples)	1,359	1,782	494,678	RMD	rs4238010	0.58	5.80 × 10^−6^	chr12:4118067

**Sullivan et al., 2009**									

Netherlands	Discovery	1,738	1,802	435,291	MD	rs2715148	0.79	7.70 × 10^−7^	chr7:82449785
Netherlands	Replication	6,079	5,893	–	–	rs2715148	NA	8.20 × 10^−1^	chr7:82449785

**Shyn et al., 2011**									

U.S.	Discovery	1,221	1,636	382,598	RMD 901; MD 735	rs12462886	0.76	1.73 × 10^−6^	chr19:29263440
U.S.	Meta	3,957	3,428	–	–	rs1106634	1.295	6.78 × 10^−7^	chr8:20065799

**Shi et al., 2011**									

U.S.	Discovery	1,020	1,636	671,421	RMD 1,000; MD 20	rs17077450	1.61	1.83 × 10^−7^	chr18:65285279

**Wray et al., 2012**									

Australia/Europe/U.S.	Discovery	2,431	3,673	1,251,157	RMD 1,145; MD 1,286	rs182358	0.78	8.80 × 10^−6^	chr1:97462900
Australia/Europe/U.S.	Meta	5,763	6,901	–	–	rs12446956	1.22	1.10 × 10^−6^	chr16:73501786

**Ripke et al., 2013b**									

Australia/Europe/U.S.	Discovery	9,240	9,519	1,235,109	RMD/MD	rs11579964	0.846	1.00 × 10^−7^	chr1:224538690
Australia/Europe/U.S.	Replication	6,783	50,695	–	–	rs1969253	1.049	4.79 × 10^−6^	chr3:183876262

**Kohli et al., 2011**									

Europe/U.S.	Discovery	353	366	365,676	MD/RMD	rs1545843	2.84	5.53 × 10^−8^	chr12:84563818
Europe/U.S.	Replication	3,738	10,635	–	–	rs1545843	1.315	1.40 × 10^−9^	chr12:84563818

**Rietschel et al., 2010**									

German	Discovery	604	1,364	491,238	MD	rs2765493	1.45	2.26 × 10^−7^	chr1:157797750
German	Meta	1,013	1,905	–	–	rs7713917	0.75	1.48 × 10^−6^	chr5:78828999

This table gives the number of cases and controls for each GWAS and summarizes results. The sample sizes listed are those used in the discovery phase, replication, and meta-analyses (meta). The number of SNPs given is that used in the association analysis, which in some cases (Wray et al., 2012, Ripke et al., 2013b) includes imputed data. The highest scoring markers are listed for each study, with their odds ratio (OR) and chromosomal location. Studies used different inclusion criteria; these are summarized under the column headed phenotype, in which “RMD” is recurrent major depression and “MD” is major depression. Where provided, the numbers of each phenotypic category are listed.

A simple summary of Table 1 is that nothing significant has been found and indeed many of the papers and reviews of this field make that point (e.g., Cohen-Woods et al., 2013). However, one paper claims a genome-wide significant association: a marker within a gene desert on chromosome 12 (Kohli et al., 2011). We need to consider not only whether this finding is likely to be true, but also whether the negative findings are meaningful. In short, how do we assess false-positive and false-negative rates in Table 1?

Interpreting the results presented in Table 1 requires an understanding of what GWAS detects. GWAS interrogates common variation in the genome, usually variants with frequencies greater than 5%, and typically requires a genome-wide significance threshold of 5 × 10^−8^ (Pe’er et al., 2008) (this threshold depends on a number of factors, including the number of variants tested, also listed in Table 1). For the diallelic SNPs that are genotyped on GWAS arrays, allele frequencies are usually reported as the frequency of the least common allele (which will always be <0.5). This is the minor allele frequency (MAF).

Genotypes from dense sets of SNPs are partially, and locally, correlated (Sachidanandam et al., 2001). The pattern of correlation is nonrandom, since recombination does not occur uniformly across the genome but is localized to hotspots (McVean et al., 2004), giving rise to blocks of linkage disequilibrium. The extent of linkage disequilibrium (that is to say the degree of correlation between markers) is one determinant of the ability of a set of markers on a genotyping array to detect genetic signal. An important consequence is that genotyping only a subset of loci captures most of the common variation in the genome. Conversely, if a causative variant is not correlated with any markers on a genotyping array, it cannot be detected. The degree to which genotyping arrays capture genomic information is partly population specific, because population history affects the extent of linkage disequilibrium. Thus, linkage disequilibrium tends to increase the further away a population is from Africa (Conrad et al., 2006), consistent with the hypothesis that humans migrated out of Africa, experiencing severe population bottlenecks on the way to colonizing the rest of the world (DeGiorgio et al., 2009, Jakobsson et al., 2008). Standard GWAS approaches do not work so well in African populations (Teo et al., 2010).

**Figure 1:**
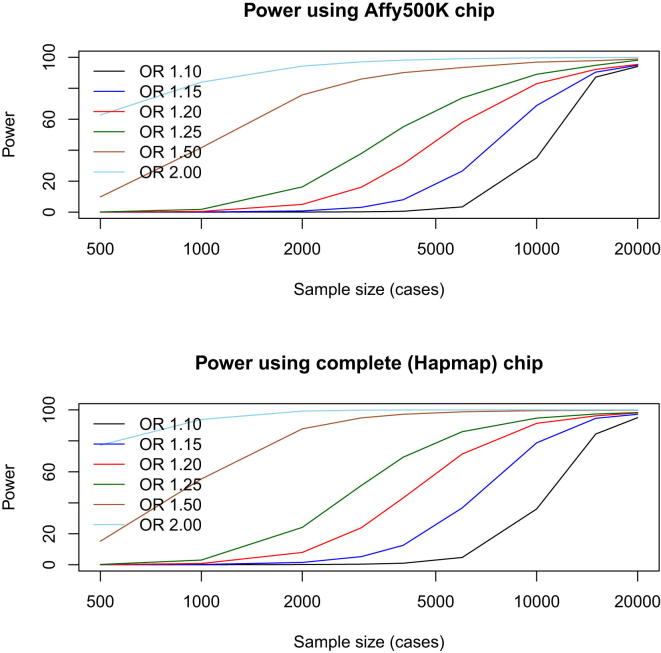
Power to Detect a Locus using GWAS Simulated data are plotted to assess power under two different sets of conditions: varying the size of the locus effect (expressed as an odds ratio [OR]) and varying the number of loci used in the GWASs. Results are shown for an Affymetrix 500K array (which yields approximately 400,000 useful genotypes per individual) and simulations using all variants in HapMap (release 2). The simulations are taken from Spencer et al. (2009). The significance level was set at 5 × 10^−8^. Sample size is shown for the number of cases required; the simulations assume an equal number of controls.

One explanation for the failure of GWAS applied to MD might be that the causative variants, or markers sufficiently close to them, have not been genotyped on the available arrays. In fact, due to the blocks of linkage disequilibrium, in non-African populations GWAS is remarkably effective at detecting a large fraction of common variants of reasonable effect size (odds ratios greater than 1.2) that contribute to complex traits, even though a very small fraction of the total amount of sequence variation segregating in a population is actually genotyped. To illustrate this, Figure 1 shows the results of simulations that compare GWAS carried out using an Affymetrix 500K genotyping array, with the results from using all the variants in HapMap (Frazer et al., 2007). Even this relatively sparse array (current platforms interrogate millions of variants) has power of 82% (for a sample size of 9,000) to detect a locus with an odds ratio of ≥1.2, compared to 88% with the complete set of SNPs (9,240 is the largest discovery sample size used in GWAS of MD [Ripke et al., 2013b]). In other words, differences in coverage between chips do not translate into big differences in power. Furthermore, imputation (Howie et al., 2009) using the very high density of variants available from the 1000 Genomes Project (Abecasis et al., 2010), has further extended the scope of genotyping arrays to interrogate millions of ungenotyped variants. In short, failure of GWAS to detect common variants (MAF > 5%) conferring risk to MD is unlikely to be due to insufficient information about these variants from genotyping arrays.

The most likely explanation for the failure of GWAS for MD is that studies have been underpowered to detect the causative loci (Wray et al., 2012). While GWAS coverage of common variants is good, GWAS requires large sample size in order to obtain adequate power to detect variants of small effect (odds ratios less than 1.2). In the following sections, we treat with common variants and the power of GWAS (and candidate gene studies) to find them. We turn later to the detection of rare variants of larger effect.

Figure 1 demonstrates the nonlinear relationship between sample size and effect size for common variants. To detect loci with an odds ratio of 1.1 or less, sample sizes in the tens of thousands will be required (note that this depends on the prevalence of the disease; in the following discussions, we assume that MD has a prevalence of 10%). Table 1 shows that the largest GWAS for MD used 9,240 cases and 9,519 controls (Ripke et al., 2013b). Figure 1 shows that such a sample has ∼90% power to detect loci with an odds ratio of ≥1.2; it will detect effects of this magnitude or greater at more than 93% of all known common variants. Note that the one positive finding reported in Table 1 is an outlier: no other GWAS detected the signal (Kohli et al., 2011). The study used a discovery sample of 353 cases and 366 controls to detect, at genome-wide significance, an association between MD and a marker next to the *SLC6A15* gene (Kohli et al., 2011). Without further replication, the status of this finding is dubious and is likely to be a false positive.

While Table 1 only includes GWASs of MD, there are also a number of studies of phenotypes that are genetically related to MD, such as the personality trait of neuroticism (Kendler et al., 1993, Shifman et al., 2008) or depressive symptoms (Foley et al., 2001, Hek et al., 2013). These studies are also negative. The largest is a study of depressive symptoms in 34,549 individuals that reports one, unreplicated, p value of 4.78 × 10^−8^. Overall, we can conclude that no study has robustly identified a locus that exceeds genome-wide significance for MD or genetically related traits. We can also conclude that GWAS results have set some constraints on the effect sizes likely to operate at common variants contributing to susceptibility to MD.

### Candidate Genes

Candidate gene studies of MD have generated many publications but few robust findings. At the time of writing (2013), searching for articles dealing with genetic association and MD returned more than 1,500 hits. Almost 200 genes have been subject to testing, many by multiple groups (Bosker et al., 2011, López-León et al., 2008). The difficulty, common in this area of research, is that few groups agree with each other. Resolution of conflicting results is usually attempted through meta-analysis and Table 2 summarizes data for 26 genes analyzed by meta-analysis, of which seven yield a significant (p < 0.05) result: *5HTTP/SLC6A4*, *APOE*, *DRD4*, *GNB3*, *HTR1A*, *MTHFR*, and *SLC6A3*.

**Table 2:** Candidate Gene Meta-analyses

Reference	Number of Studies	Number of Cases	Number of Controls	p Value	OR	95% CI	Variant	MAF	Power	Number for 80% Power
**5-HTR2A**										

Anguelova et al., 2003	7	768	959	0.597	0.96	0.84–1.11	rs6311	0.44	6.2%	55,781
Jin et al., 2013	11	1,491	2,937	0.12	NA	NA	rs6311	0.44	NA	NA

**5HT-6R**										

Fukuo et al., 2010	4	701	2,422	0.406	0.94	0.80–1.08	rs1805054	0.17	8.4%	19,021

**5HTTP/SLC6A4**										

Clarke et al., 2010	39	6,836	14,903	0.007	1.09	1.02–1.16	44 bp ins/del	0.43	65.8%	9,575
Anguelova et al., 2003	11	941	2,110	0.198	1.08	0.96–1.22	44 bp ins/del	0.43	12.3%	11,958
Anguelova et al., 2003	10	592	2,094	>0.5	NA	NA	intron 2 VNTR	0.35	NA	NA
Furlong et al., 1998	4	275	739	0.049	1.2	1.00–1.45	44 bp ins/del	0.43	17.3%	2,112
Lasky-Su et al., 2005	14	1,961	3,402	0.28	1.05	0.96–1.14	44 bp ins/del	0.43	10.5%	32,911
López-León et al., 2008	22	3,752	5,707	<0.05	1.11	1.04–1.19	44 bp ins/del	0.43	51.6%	7,356
López-León et al., 2008	8	NA	NA	NS	1.33	0.78–2.27	intron 2 VNTR	0.35	NA	NA

**ACE**										

Wu et al., 2012	15	2,479	7,744	NS	1.15	1.02–1.3	Ins/del intron 16	0.45	65.9%	3,465
López-León et al., 2006	4	586	5,169	>0.1	0.85	0.55–1.3	Ins/del intron16	0.45	33%	1,992
López-León et al., 2008	8	NA	NA	NS	1.08	0.97–1.2	Ins/del intron16	0.45	NA	NA

**BDNF**										

Gyekis et al., 2013	3	331	688	0.103	0.83	0.67–1.04	rs16917204	0.24	20.7%	2,001
Gyekis et al., 2013	2	285	746	0.527	1.16	0.74–1.82	rs2030324	0.46	22.5%	2,340
Gyekis et al., 2013	2	777	1,541	0.831	0.98	0.85–1.14	rs988748	0.26	5.4%	193,287
Gyekis et al., 2013	23	4,173	12,747	0.402	0.96	0.89–1.05	rs694	0.43	14.1%	43,058
Chen et al., 2008b	9	3,879	3,151	0.918	1	0.94–1.07	rs694	0.43	NA	NA
López-León et al., 2008	8	NA	NA	NS	1.01	0.93-1.09	rs694	0.43	NA	NA
Verhagen et al., 2010	14	2,812	10,843	>0.1	1.06	0.94–1.19	rs694	0.43	19.5%	18,385

**CLOCK**										

Kishi et al., 2011	6	930	2,305	0.47	0.95	0.83–1.09	rs1801260	0.22	8.4%	25,381

**COMT**										

López-León et al., 2008	6	NA	NA	NS	0.98	0.86–1.13	rs4680	0.39	NA	NA

**DRD3**										

López-León et al., 2008	4	541	606	NS	1.06	0.85–1.34	rs6280	0.45	13.8%	5,721

**DRD4**										

López León et al., 2005	5	318	814	0.003	1.73	1.29–2.32	48 bp ins/del	0.45	95.6%	185

**GABRA3**										

López-León et al., 2008	6	NA	NA	NS	0.91	0.68–1.2	CA repeat intron 8	0.29	NA	NA

**GNB3**										

López-León et al., 2008	3	375	492	<0.05	1.38	1.13–1.69	rs5443	0.48	51.2%	743

**HTR1A**										

Kishi et al., 2009	7	1,658	2,046	0.0327	0.821	0.695–0.984	rs6295	0.48	65.9%	2,315
López-León et al., 2008	4	NA	NA	NS	1.16	0.98–1.38	rs6295	0.48	NA	NA
Kishi et al., 2013	13	3,199	4,380	0.006	0.87	0.78–0.96	rs6295	0.48	61.9%	4,901

**HTR1B**										

López-León et al., 2008	3	NA	NA	NS	0.96	0.77–1.2	rs6296	0.35	NA	NA

**HTR2A**										

Gu et al., 2013	4	780	1,528	0.1	0.91	0.74–1.12	rs6311	0.44	13.9%	8,229
López-León et al., 2008	4	NA	NA	NS	1.01	0.85–1.21	rs6311	0.44	NA	NA
López-León et al., 2008	8	NA	NA	NS	0.96	0.84–1.09	rs6313	0.43	NA	NA

**HTR2C**										

López-León et al., 2008	2	NA	NA	NS	1.03	0.85–1.25	rs6318	0.17	NA	NA

**MAOA**										

López-León et al., 2008	4	NA	NA	NS	0.86	0.65–1.13	VNTR promoter	0.34	NA	NA

**MTHFR**										

Peerbooms et al., 2011	17	3,341	13,840	0.579	1.016	0.96–1.07	rs1801133	0.32	6.2%	250,625
Lewis et al., 2006	9	1,241	1,1021	0.003	1.36	1.11–1.67	rs1801133	0.32	99.1%	518
Gilbody et al., 2007	10	1,280	10,429	<0.05	1.14	11.04–1.26	rs1801133	0.32	43.4%	3,121
Zintzaras, 2006	5	291	897	>0.1	1.15	0.97–1.36	rs1801133	0.32	13.4%	3,223
López-León et al., 2008	6	875	3,859	<0.05	1.2	1.07–1.34	rs1801133	0.32	51.5%	1,719
Gaysina et al., 2008	4	1,222	835	0.39	0.96	0.84–1.09	rs1801133	0.32	6.4%	80,906

**NET/SLC6A2**										

Zhao et al., 2013	6	1,673	1,410	0.78	1.02	0.91−1.13	rs5569	0.27	5.5%	281,312
Zhao et al., 2013	6	1,681	2,938	0.78	1.03	0.84−1.27	rs2242446	0.26	6.7%	90,329
López-León et al., 2008	3	NA	NA	NS	0.97	0.8–1.18	rs2242446	0.26	NA	NA

**DAT/SLC6A3**										

López-León et al., 2008	3	151	272	<0.05	2.06	1.25–3.4	VNTR 3-UTR	0.48	90.1%	112
López-León et al., 2008	3	NA	NA	NS	0.94	0.84–1.05	VNTR 3-UTR	0.48	NA	NA

**TH (TPH1)**										

Chen et al., 2008a	10	1,812	2,223	>0.1	NA	NA	rs1800532	0.36	NA	NA

**TPH1**										

López-León et al., 2008	9	NA	NA	NS	0.88	0.71–1.09	rs1800532	0.36	NA	NA

This table summarizes the data from meta-analyses of candidate genes in which variants have been tested for association with major depression (MD). The table is sorted by gene to allow comparison between studies of the same gene. Note that some studies test different variants within the same gene. The column headed “Variant” gives the variant tested. Throughout the table, “NA” means “not available” and NS “nonsignificant.” The table gives sample sizes for the number of studies included in the meta-analysis (Number of Studies), the total number of cases and controls (Number of Cases and Number of Controls), the p value (where available), the odds ratio (OR), and associated 95% confidence interval (95% CI). Where the variant is an SNP, an rs number is provided along with the minor allele frequency (MAF) in European populations. For repeats, the frequency of the commonest variant is given. The power of each study, expressed as a percentage (Power) was calculated from the odds ratio of the meta-analysis, using the Genetic Power Calculator (Purcell et al., 2003). Power was calculated assuming an additive model and with marker allele frequencies set to 0.5 (a conservative assumption).

We can use the results from Table 1 to interpret the results presented in Table 2. First, we note that the mean effect size (expressed as an odds ratio) across the studies that report a significant effect is 1.35. Second, all of the variants tested, whether significant or not, are common; none have an MAF less than 10%, and the mean is 38% (column headed MAF in Table 2). This means that the results of GWAS are relevant (recall that GWAS interrogates common variants). Virtually all of the candidate variants should be detectable by the published GWAS, particularly if imputation is used to obtain data from markers not present on the arrays (Howie et al., 2009) (Figure 1). The fact that the candidate variants do not occur in Table 1 suggests that the results in Table 2 are false positives (recall that the largest published GWAS has greater than 80% power to detect an odds ratio greater than 1.2).

Most GWASs include a section reporting the analysis of variants in candidate genes, and by providing a much larger sample size than almost any of the meta-analyses listed in Table 2, their findings are likely to be more robust than the meta-analyses. Boomsma and colleagues tested 92 SNPs in 57 candidate genes in a GWAS sample of 1,738 cases and 1,802 controls (Bosker et al., 2011). Two SNPs (in *C5orf20* [Willis-Owen et al., 2006] and in *NPY* [Heilig et al., 2004]) scored p values less than 0.05, where four would have been expected by chance. The finding is therefore compatible with no effect at any locus tested. At the gene level (testing for enrichment of significant SNPs), two genes passed the 5% threshold, *TNF* (Jun et al., 2003) and the norepinephrine transport (*NET*) (Inoue et al., 2004), again compatible with chance expectations. Wray and colleagues tested 180 candidate genes, and after correcting for the number of tests carried out, found that no candidate gene was significantly associated (Wray et al., 2012).

Table 2 shows the power of each meta-analysis, using the effect size estimated from each meta-analysis (Purcell et al., 2003) and assuming a disease prevalence of 10%. The mean odds ratio estimated over all candidate gene meta-analyses is 1.15, requiring a sample size of greater than 3,000 cases. Only six meta-analyses use sample sizes in excess of 3,000, and just two of these six reported a significant finding. Note that for those studies reporting a significant result, the mean power was only 60%.

In summary, the data from Table 2 are consistent with a lack of significant findings in any candidate gene meta-analysis. Moreover, the meta-analyses discussed here represent less than a quarter of all the genes tested in the literature (and a smaller fraction of the variants). With lower sample sizes than reported in the meta-analyses, the findings for individual genes are weaker than for those reported in Table 2.

However, lack of evidence does not mean an effect can be excluded; the negative findings are also compatible with a lack of power to detect an effect. In fact, as we discuss below, estimates of the likely number of genetic variants contributing to MD risk run into the thousands. Given that about 18,000 genes are expressed in the brain (Lein et al., 2007), it would not be surprising if some of the candidates in Table 2 are true risk variants, but nowhere near the effect size currently considered plausible. This raises the question, so far unanswered, at what point can we say a candidate has been excluded.

Nevertheless the conclusion is straightforward: candidate gene studies provide little convincing support for the involvement of any candidate gene in MD. This point should be born in mind by all those wishing to use association data to support a particular explanation of the biological causes of depression. Neuroscientists sometimes claim that genetic results can be interpreted as evidence in favor of their particular theory (Duman et al., 1997, Holsboer, 2000, Luscher et al., 2011, Samuels and Hen, 2011). Any such claims should be treated with extreme caution.

### The Contribution of Common Variants to Disease Risk

GWAS data can be used to constrain further the likely genetic architecture of MD, by using marker results that do not reach genome-wide significance. This is important because it might be that the genetic architecture of MD consists primarily of rare but relatively large effect loci. For example, it could be that there are many susceptibility alleles with frequencies much less than 5% and odds ratios greater than 3. Nothing we have so far said has excluded this possibility. However, GWAS results make that extremely unlikely, as can be appreciated from the following argument.

Suppose that the genetic architecture of MD consists of many small-effect loci, smaller than can be detected at genome-wide significance by currently available samples. For example, suppose the odds ratio for these risk variants are 1.05 and suppose the variants have a frequency of 50% (alleles with a higher frequency are easier to detect, so this is a conservative assumption). Power to detect a single variant of this effect size at this frequency in a sample size of 10,000 cases and 10,000 controls is less than 0.001%, at a p value of 1 × 10^−7^ and disease prevalence of 10% (Purcell et al., 2003). But there is a 67% chance that such a variant will have a p value less than 0.5. This means that if all SNPs are ranked by their p values, then p values less than 0.5 will be enriched with SNPs that contribute to disease susceptibility. In other words, if there are small-effect variants contributing to MD, then the distribution of SNP p values will depart from null expectations. This method is referred to as polygenic scoring and has been used to investigate the polygenic nature of complex traits.

A second class of method uses the SNP data to estimate genetic similarity and thereby assess heritability. GWAS SNPs are common variants, shared by descent from common ancestors. Regions of the genome contributing to disease susceptibility will be enriched among those with the same disease. The degree of sharing of common variants will reflect the heritability of the trait, at least that portion due to such common variation. Thus, by assessing the amount of sharing by descent between individuals with the disease, it is possible to estimate the heritability from SNPs (hence sometimes called SNP heritability). There are currently two implementations of this idea (So et al., 2011, Yang et al., 2011).

Two papers report SNP heritabilities for MD ranging from 21% (Lee et al., 2013) to 30% (Lubke et al., 2012). The discrepancy between SNP- and family-based heritability estimates (of about 38%) is in part attributable to the fact that causal variants are not in linkage disequilibrium with genotyped markers (Yang et al., 2010a); this means that the SNP-based heritability is a lower bound on that arising from common variants.

Even though the SNP heritabilities have wide confidence intervals (from 15%–50%), they provide a critically important constraint on our understanding of the genetics of MD: they indicate that common variants of small effect (with odds ratio less than 1.2, and probably much less) make a large contribution to the genetic susceptibility to the disease, accounting for more than 50% of the heritability. Indeed, the SNP heritability is consistent with the view that the genetic basis of MD consists of many thousands of independently acting loci, each of very small effect, that contribute to disease susceptibility. Before we consider some alternative possibilities, we pursue what this conclusion means for genetic studies of MD. What is needed to find robust, genome-wide significant association? Can we estimate the sample size needed?

**Figure 2:**
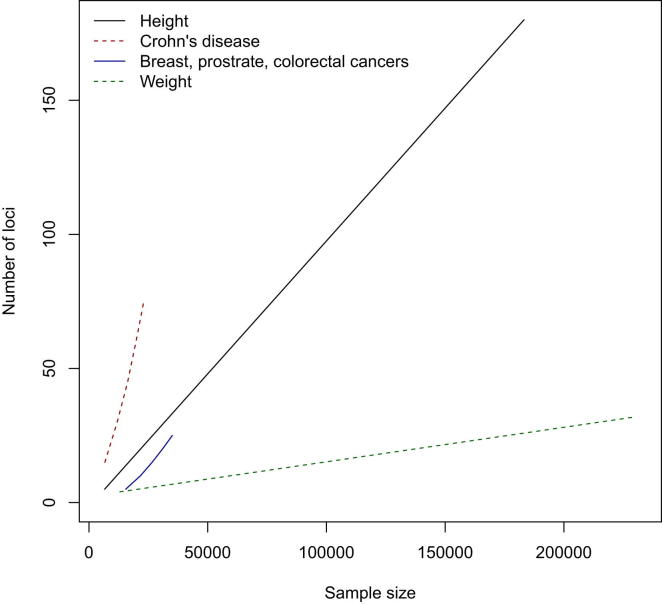
GWAS Sample Size Sample sizes (horizontal axis) required for a GWAS to have 80% probability of detecting the number of loci shown on the vertical axis, at a significance level of 5 × 10^−8^. Results are shown for four complex traits: two disease and two quantitative phenotypes. The graph assumes that the number of loci detected increases linearly with increasing sample size (data are from Frayling et al., 2007, Lango Allen et al., 2010, Loos et al., 2008, Park et al., 2010, Scuteri et al., 2007, Speliotes et al., 2010, Thorleifsson et al., 2009, Wen et al., 2012, Willer et al., 2009).

Complex traits show clear differences in the number of samples required to obtain a significant finding. Figure 2 shows results for two diseases (cancer and Crohn’s disease) and two quantitative traits (height and weight) (Park et al., 2010). Which genetic architecture is most similar to that of MD? If we could answer this question, we would be in a good position to estimate the sample sizes needed to detect genetic loci, thus informing our interpretation of existing data, and the design of future experiments.

Wray and Visscher asked this question about the genetic architecture of schizophrenia (Wray and Visscher, 2010). Their answer involved finding a phenotype with a genetic architecture predicted to be similar to schizophrenia and for which many genetic loci have been found. They suggested, from similar heritability estimates, risks to relatives, and the disease prevalence, that the genetic architecture of schizophrenia resembles that of height. In order to compare genetic analysis of height with schizophrenia, they assume that genetic liability to schizophrenia is quantitative and that the dichotomous nature of schizophrenia arises because the number of predisposing alleles in some individuals exceeds a certain threshold. For example, an individual with predisposing alleles at 100 loci or more might present with schizophrenia, while someone with fewer such alleles would show no symptoms. By considering that disease prevalence represents the fraction of individuals whose genetic susceptibility exceeds this threshold, and that schizophrenia has otherwise the same genetic architecture as height, it is possible to apply what we know from height GWAS data to estimate sample sizes needed to detect schizophrenia risk loci (Yang et al., 2010b).

In order to compare the power to detect a locus affecting a disease in a case-control study with the power to detect a locus affecting a quantitative trait (assuming that both have the same genetic architecture and heritability), Visscher and colleagues show that only the disease prevalence and proportion of cases and controls need be known (Yang et al., 2010b). This means that we can estimate sample sizes for a GWAS of MD by comparing it with a quantitative trait that has a similar genetic architecture and for which loci have been found. But which quantitative trait is appropriate?

**Figure 3:**
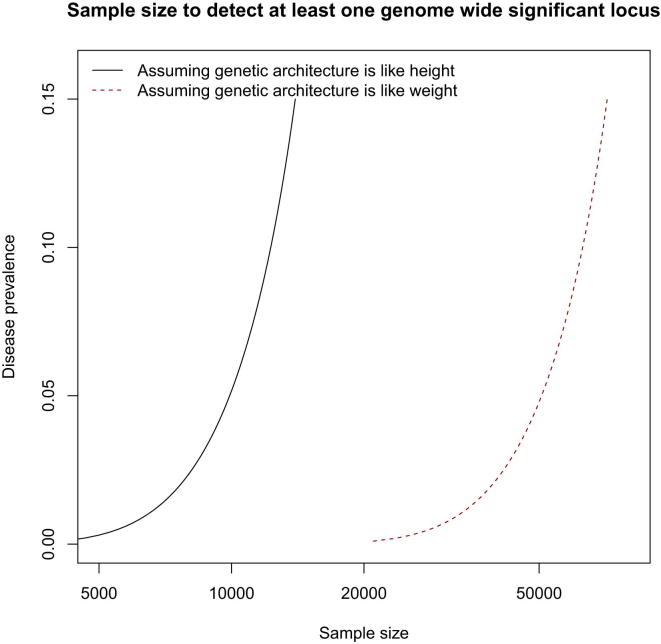
The Effect of Disease Prevalence on Sample Size to Detect at Least One Locus for Major Depression Sample sizes (horizontal axis) required for a GWAS to have 80% probability of detecting at least one locus contributing to the risk of major depression, plotted against disease prevalence (vertical axis). In most surveys, major depression has a prevalence of about 10%. The genetic architecture of major depression is assumed to be either similar to height (black continuous line) or weight (red dotted line).

Weight (or more properly body mass index) might be an appropriate model: many loci have been mapped (Berndt et al., 2013, Speliotes et al., 2010) and it has a heritability similar to MD (a recent estimate based on 20,000 sibling pairs gave 40% [Hemani et al., 2013], though this is lower than a large meta-analysis of twin data [Nan et al., 2012]). In Figure 3, we show results under the assumption that MD has a similar genetic architecture to weight (red dotted line) or to height (black continuous line) (Yang et al., 2010b). We estimated the number of samples needed for an MD GWAS to have 80% power to detect at least one locus, for different disease prevalences.

If MD has a genetic architecture similar to weight (red dotted line), then, for a disease prevalence of 10% (typical of most surveys of MD), a sample size of more than 50,000 cases will be needed to detect at least one genome-wide significant hit. About 10,000 cases are needed if MD has a genetic architecture similar to height. Figure 3 also shows that disease prevalence has a big impact on power. For example, while power to detect a variant that explains 0.08% of the variance on liability to MD will be 4%, in a sample size of 10,000 cases and 10,000 controls, power in schizophrenia (prevalence 1%) is approximately 50% for the same sample size.

The effect of disease prevalence (shown on the vertical axis) is not linearly related to sample size. In order to find genes with a smaller sample size, we need to collect a sample that has a lower prevalence. That could be achieved in one of two ways. If MD is truly a quantitative phenotype, then the extremes of the distribution will represent a less prevalent form of disease. We could take disease that is so severe that it has a prevalence of 0.5% or lower, so that fewer than 20,000 cases would provide 80% power to detect at least one locus. The problem is finding the appropriate severity scale.

Alternatively, we could identify rare subtypes of depression that are less prevalent and we hope represent a more homogenous condition than MD broadly defined. Ideally, such subtypes would have a different genetic architecture, veering more toward that of height than of weight, so that much smaller samples are needed. Do such heritable subtypes of MD exist? We address this question below. We start however with a review of the genetics literature to determine whether there might be rare but relatively large-effect loci that GWASs have been unable to detect.

### The Contribution of Rare Variants to Disease Risk

The data we have summarized so far are compatible with the hypothesis that the genetic basis of MD arises from the joint effect of very many loci of small effect, with odds ratios of much less than 1.2. However, it is also compatible with the existence of larger effect loci, under two alternative (but not incompatible) hypotheses; first, some of the heritability of MD is explained by rare relatively large-effect loci; second, larger effect sizes would be observed if more homogeneous heritable phenotypic groupings could be identified. We consider in this section whether rare, large-effect variants might exist and return to the issues of phenotypic homogeneity later.

It is sometimes forgotten that linkage studies provide information about rare, relatively penetrant susceptibility loci. Family-based designs are typically not well powered to detect the small effects found in GWASs. For example, on average, siblings share 50% of their genome. Where two siblings have the same disease, departure from this 50% sharing indicates regions that harbor risk variants; but since the SD for sharing is large (approximately 3.7%), large sample sizes are required to detect a significant departure. Family designs can however detect one form of genetic variation that is hidden from GWASs: the joint effect of independent, rare, mutations in the same gene (recall that GWASs are effective for common variants). In a linkage study, the effects of independent mutations will combine together, since the unit of analysis in linkage (the average distance between recombinations in the human genome in a single meiosis) is a much larger genomic region than is the case for association analyses. In cases in which linkage asserts that there is an effect but association fails to detect one, then one explanation is allelic heterogeneity: multiple effects exist in the gene but on different haplotypes.

Linkage studies are summarized in Table 3. Results are reported as a logarithm of the odds (LOD) score, rather than a p value. The majority of the studies reported in Table 3 used an affected sibling design (in which two siblings have MD). In this design, an LOD score of 2.2 is suggestive evidence for linkage (expected to occur once by chance in a genome scan), an LOD score greater than 3.6 represents significant linkage (expected to occur by chance with a probability of 5%), and an LOD score of 5.4 is highly significant (probability of chance occurrence is less than 0.1%) (Lander and Kruglyak, 1995).

**Table 3:** Linkage Studies

Phenotype	Study Name	Families	Individuals	%F	Clinical Instrument	Peak Marker	Peak LOD Score	Marker Location	Sex
**Zubenko et al., 2003**									

RMD 375 pairs; MD 520 pairs; mood disorder 610 pairs; depression spectrum 520	Pittsburgh families	81	1,242	51.63	SADS-L	D1S1597	3.6	chr1:13,684,108-13,884,418	–
RMD 375 pairs; MD 520 pairs; mood disorder 610 pairs; depression spectrum 520	Pittsburgh families	81	1,242	51.63	SADS-L	D1S1609	2.7	chr1:243,965,857-244,166,112	–
RMD 375 pairs; MD 520 pairs; mood disorder 610 pairs; depression spectrum 520	Pittsburgh families	81	1,242	51.63	SADS-L	D2S427	2.77	chr2:232,106,263-232,306,614	–
RMD 375 pairs; MD 520 pairs; mood disorder 610 pairs; depression spectrum 520	Pittsburgh families	81	1,242	51.63	SADS-L	D5S1503	3.32	chr5:98,071,660-98,272,056	–
RMD 375 pairs; MD 520 pairs; mood disorder 610 pairs; depression spectrum 520	Pittsburgh families	81	1,242	51.63	SADS-L	D5S1505	3.74	chr5:119,001,596-119,201,988	–
RMD 375 pairs; MD 520 pairs; mood disorder 610 pairs; depression spectrum 520	Pittsburgh families	81	1,242	51.63	SADS-L	D8S1477	1.74	chr8:31,966,957-32,167,504	–
RMD 375 pairs; MD 520 pairs; mood disorder 610 pairs; depression spectrum 520	Pittsburgh families	81	1,242	51.63	SADS-L	D10S1221	3.01	chr10:57,429,886-57,630,151	–
RMD 375 pairs; MD 520 pairs; mood disorder 610 pairs; depression spectrum 520	Pittsburgh families	81	1,242	51.63	SADS-L	D10S2470	2.61	chr10:92,264,596-92,464,872	–
RMD 375 pairs; MD 520 pairs; mood disorder 610 pairs; depression spectrum 520	Pittsburgh families	81	1,242	51.63	SADS-L	D11S1984	4.2	chr11:1,466,686-1,667,029	–
RMD 375 pairs; MD 520 pairs; mood disorder 610 pairs; depression spectrum 520	Pittsburgh families	81	1,242	51.63	SADS-L	D11S2002	2.1	chr11:79,865,382-80,065,662	–
RMD 375 pairs; MD 520 pairs; mood disorder 610 pairs; depression spectrum 520	Pittsburgh families	81	1,242	51.63	SADS-L	D15S1012	1.96	chr15:38,907,527-39,107,917	–
RMD 375 pairs; MD 520 pairs; mood disorder 610 pairs; depression spectrum 520	Pittsburgh families	81	1,242	51.63	SADS-L	D18S858	2.93	chr18:54796986-54997312	–
RMD 375 pairs; MD 520 pairs; mood disorder 610 pairs; depression spectrum 520	Pittsburgh families	81	1,242	51.63	SADS-L	D19S586	2.49	chr19:9,704,793-9,905,143	–

**Abkevich et al., 2003**									

RMD 784; MD 161; BPD 162	Utah families	110	1,357	68.98	BPS	D12S1600	6	chr12:99,200,748-99,401,155	Male

**Camp et al., 2005**									

RMD 1,513; anxiety 1,141, of which only 718 used	Utah families	87	NA	NA	–	D3S1752	3.81	chr3:97,645,283-97,845,588	–
RMD 1,513; anxiety 1,141, of which only 718 used	Utah families	87	NA	NA	–	D7S517	2.89	chr7:4,397,915-4,598,292	–
RMD 1,513; anxiety 1,141, of which only 718 used	Utah families	87	NA	NA	–	D18S1270	3.75	chr18:61,292,502-61,492,816	–
RMD 1,513; anxiety 1,141, of which only 718 used	Utah families	87	NA	NA	–	D15S515	2.88	chr15:43,497,354-43,697,543	Male
RMD 1,513; anxiety 1,141, of which only 718 used	Utah families	87	NA	NA	–	D4S2631	2.6	chr4:155,863,840-156,064,129	Male

**McGuffin et al., 2005**									

RMD 929	DeNt	417	929	70.94	SCAN	D1S450	3.03	chr1:9,485,419-9,685,791	Female
RMD 929	DeNt	417	929	70.94	SCAN	D12S1613	1.57	chr12:107,538,542-107,738,864	–
RMD 929	DeNt	417	929	70.94	SCAN	D13S170	1.47	chr13:81,009,094-81,209,378	–
RMD 929	DeNt	417	929	70.94	SCAN	D1S2667	2.54	chr1:11,386,961-11,587,307	Female
RMD 929	DeNt	417	929	70.94	SCAN	D1S508	2.19	chr1:7,507,384-7,707,656	Female
RMD 929	DeNt	417	929	70.94	SCAN	D12S1683	1.29	chr12:106,085,479-106,285,844	–
RMD 929	DeNt	417	929	70.94	SCAN	D15S1047	1.14	chr15:81,041,079-81,241,425	–
RMD 929	DeNt	417	929	70.94	SCAN	D15S999	1.08	chr15:86,145,362-86,345,589	–

**Breen et al., 2011**									

RMD 2,164	DeNt	NA	2,412	72.43	SCAN	D3S1515	4.01	chr3:6,311,334-6,511,566	–
RMD 2,164	DeNt	NA	2,412	72.43	SCAN	D7S513	1.91	chr7:11,551,237-11,751,614	–
RMD 2,164	DeNt	NA	2,412	72.43	SCAN	D11S937	1.75	chr11:77,754,318-77,954,608	–
RMD 2,164	DeNt	NA	2,412	72.43	SCAN	D10S1653	1.6	chr10:15,577,832-15,778,169	–
RMD 2,164	DeNt	NA	2,412	72.43	SCAN	D1S450	0.75	chr1:9,485,419-9,685,791	–
RMD 2,164	DeNt	NA	2,412	72.43	SCAN	D12S1613	<1	chr12:107,538,542-107,738,864	–
RMD 2,164	DeNt	NA	2,412	72.43	SCAN	D15S999	1.41	chr15:86,145,362-86,345,589	–
RMD 2,164	DeNt	NA	2,412	72.43	SCAN	D13S170	<1	chr13:81,009,094-81,209,378	–

**Holmans et al., 2004**									

RMD 809	GenRED	297	1,039	79.00	DIGS	D15S652	3.73	chr15:92,417,335-92,617,665	–

**Holmans et al., 2007**									

RMD 1,720; MD 28	GenRED	656	2,176	79.63	DIGS	D15S652	3.05	chr15:92,417,335-92,617,665	–
RMD 1,720; MD 28	GenRED	656	2,176	79.63	DIGS	D17S974	4.77	chr17:10,418,666-10,618,972	Male
RMD 1,720; MD 28	GenRED	656	2,176	79.63	DIGS	D8S1106	3.49	chr8:12,735,859-12,936,149	Male

**Levinson et al., 2007**									

RMD 1,687	GenRED	631	2,161	79.00	DIGS	NA	4.69	chr15:92,600,000	–

**Pergadia et al., 2011**									

MD and smoking 220	Australian/Finland	116	810	56.79	CIDI	D3S1304	4.14	chr3:6,819,242-7,019,583	–

**Middeldorp et al., 2009**									

MD	Australian/Dutch	133	558	61.12	CIDI	ATA58E08	2.1	chr17:19,426,481-19,426,503	–
MD	Australian/Dutch	133	558	61.12	CIDI	D8S504	1.9	chr8:917,443-1,117,767	–
MD	Australian/Dutch	133	558	61.12	CIDI	GATA66D01	1.7	chr2:66,951,054-67,151,286	–

**Schol-Gelok et al., 2010**									

Symptoms of MD 115	Dutch ERF	45	1,144	71.3	HADS-D and CES-D	rs715271	0.93	chr2:57133160	–
Symptoms of MD 115	Dutch ERF	45	1,144	71.3	HADS-D and CES-D	rs890478	0.99	chr2:64540177	–
Symptoms of MD 115	Dutch ERF	45	1,144	71.3	HADS-D and CES-D	rs372169	2.14	chr5:3180951	–
Symptoms of MD 115	Dutch ERF	45	1,144	71.3	HADS-D and CES-D	rs1965277	2.27	chr11:134850365	–
Symptoms of MD 115	Dutch ERF	45	1,144	71.3	HADS-D and CES-D	rs1688128	2.66	chr19:3029918	–

The table summarizes information from linkage studies of major depression. Most studies are represented by more than one publication (reporting additional data, or more in-depth analyses), so the second column provides a study name to indicate which studies report on the same data sets. Studies used different inclusion criteria; these are summarized under the column headed phenotype where RMD is recurrent major depression, MD is major depression, BPD is bipolar disease. Where provided, the numbers of each phenotypic category are listed. The table gives the acronym of the clinical instrument used, the peak marker, and associated LOD score for nonparametric linkage (some studies also report parametric results [Schol-Gelok et al., 2010]), without added covariates. In cases where a significant sex difference was found, this is reported in the column headed Sex.

Table 3 makes four points. First, there is clear heterogeneity between studies. The outlier here is the Zubenko study (Zubenko et al., 2003), which reports more loci at higher levels of significance than all the others. Second, there is evidence for poor internal consistency. Three groups report data in multiple publications, usually because they acquired additional data (Utah families [Abkevich et al., 2003, Camp et al., 2005], DeNt [Breen et al., 2011, McGuffin et al., 2005], and GenRED [Holmans et al., 2004, Holmans et al., 2007, Levinson et al., 2007]). The additional samples collected by the GenRED consortium failed to confirm the 15q linkage reported in their initial paper (Holmans et al., 2004). The authors considered that the first finding might be a false positive, that the second finding might be a false negative, or that both findings were true, the difference being attributable to variation in the clinical features of the families (Holmans et al., 2007).

Third, there are overlaps in the locations identified by linkage results (Table 3). The confidence intervals for the position of loci found by linkage studies are notoriously broad (Roberts et al., 1999), so that overlaps between localizations often occur by chance. However, if we restrict analysis to a window of just 5 Mb, then five regions are repeatedly found: chromosome 11, 75–80 Mb (Breen et al., 2011, Zubenko et al., 2003), chromosome 15, 37–42 Mb (Zubenko et al., 2003, Camp et al., 2005), chromosome 15, 87–92 Mb (Breen et al., 2011, Holmans et al., 2004, Holmans et al., 2007, Levinson et al., 2007), chromosome 3, 4–9 Mb (Breen et al., 2011, Middeldorp et al., 2008), and chromosome 2, 64–68 Mb (Middeldorp et al., 2008, Schol-Gelok et al., 2010). This is partly, but not entirely, due to the large number of loci found in one study (Zubenko et al., 2003), a study that has attracted criticism (e.g., unusually low simulation-based LOD score thresholds reported for analyses without covariates [Levinson, 2006]), so we cannot come to any firm conclusions, but this result suggests that some of the signal may be true.

Finally, there is some evidence that sex differences matter. Four groups report differences in linkage results when the analysis incorporates sex as a covariate. As predicted by the twin results summarized earlier, some loci appear to be sex specific (Abkevich et al., 2003, Camp et al., 2005, Holmans et al., 2007, McGuffin et al., 2005, Zubenko et al., 2003).

One interpretation of the linkage studies is that rare but relatively penetrant variants might contribute to the genetic risk. Nevertheless, it is also possible that the linkage findings could be explained as false positives or the overinterpretation of nonsignificant results. In this respect, it is useful to consider the results of a study of weight in 20,240 siblings (from 9,570 nuclear families) showing that a highly polygenic genetic architecture (such as that underlying MD) can falsely indicate the presence of large-effect loci in a linkage analysis (Hemani et al., 2013).

There is some limited evidence from other sources that Mendelian-acting mutations give rise to MD. Attempts to fit morbid risk data to single major locus models have all been inconclusive (Gershon et al., 1976, Goldin et al., 1983, Price et al., 1985), as have been attempts to find markers that cosegregate with MD in a Mendelian inheritance pattern (Ashby and Crowe, 1978, Weitkamp et al., 1980, Wilson et al., 1989). A review of the online catalog of Mendelian disorders (OMIM) identified four single gene disorders in which MD is present as a clinical feature (Table 4). In addition (and not reported in the table), there are well-known relationships between MD and familial Cushing syndrome and Parkinson disease. The examples in Table 4 are rare, such as Perry syndrome, for which eight families are known worldwide, and typically present with additional phenotypes that would not lead them to be classified among the majority of cases of MD.

**Table 4:** Mendelian Conditions in which Major Depression Has Been Listed as a Phenotype

MIM	Name	Clinical Features	Prevalence	Inheritance	Gene
#168605	Perry sydrome	The earliest and most prominent symptom may be MD not responsive to antidepressant drugs or electroconvulsive therapy. Sleep disturbances, exhaustion, and marked weight loss are features.	Eight families in the world	Dominant	DCTN1
#314250	Dystonia 3, torsion, X-linked; DYT3	The odds ratio for overall MD was increased OR = 2.85, 95% CI = 0.56–5.14) in patients with DYT3 compared to the control group.	5.24 in 100,000 on Panay Island, Philippines	X-linked	TAF1
#128100	Dystonia 1, torsion, autosomal dominant; DYT1	Carriers of DYT1 are over four times more likely than noncarriers to exhibit recurrent MD. Relative risk of 3.62	In France, an estimated disease frequency of 0.13 in 100,000	Dominant	DYT1
#222300	Wolfram syndrome 1; WFS1	Additional clinical features include diverse psychiatric disorders	Heterozygous carriers of the Wolfram syndrome, estimated to represent approximately 1% of the United States population, are predisposed to MD.	Recessive	WFS1

The column headed MIM provides the reference number in Mendelian Inheritance in Man (http://www.omim.org).

Table 4 contains one example of a Mendelian mutation that underlies pure MD: Wolfram syndrome, a rare autosomal recessive disorder due to a mutation on the short arm of chromosome 4 (Polymeropoulos et al., 1994). The important point here is that Wolfram is a recessive condition. The disease itself (in homozygotes) is characterized by a broad spectrum of psychiatric and neurological disorders, but heterozygote carriers show a purer MD phenotype: in one report, out of 11 individuals carrying a Wolfram mutation, eight were hospitalized for major MD, significantly more than the three relatives expected if there were no association between psychiatric hospitalizations and mutations at this locus (Swift and Swift, 2005). The authors argue that “if the population frequency of wolframin mutations that predispose carriers to psychiatric illness is about 1%, with an odds ratio of 7.1, wolframin mutation carriers would be estimated to be about 7% of patients hospitalized for MD” (Swift and Swift, 2005).

Overall, we cannot rule out the possibility that rare large-effect risk alleles exist, but we also cannot extend much hope for their discovery. It is possible that risk alleles with odds ratios between 3 and 4, occurring at low frequencies (less than 5%), make a contribution to MD, but their discovery will require either a new generation of genotyping arrays, interrogating rare variants, or the deployment of population-scale sequencing.

### Genetics and the Nosology of MD

The second hypothesis to explore is the idea that larger-effect loci might be detected if MD were to be analyzed differently. For example, consider the possibility that MD is not one but two disorders that cannot be differentiated on a clinical basis alone. Suppose that 50 variants contribute to disease through one pathway (leading to one subtype of MD) and 50 to a second pathway (leading to the second subtype). Unbeknownst to investigators, a study contained equal numbers of the two subtypes. Since variation in the first pathway is irrelevant to disease susceptibility in the second subtype, the genetic effect of loci acting on one pathway is reduced by half, and power is similarly reduced. This point is not merely important in helping design genetic studies, it is critically important for their interpretation. Without knowledge of the existence of two unrelated mechanisms, it would be difficult, perhaps impossible, to interpret the results of the study. We would be left guessing whether the 100 variants represented one, two, or more mechanistic pathways.

Do subforms of genetically homogeneous MD exist? A large literature addresses this issue, not all of it readily summarized; here we tackle two questions that are key to understanding how genetic effects operate in MD: first, how separate is MD from other disorders? Second, is MD one disorder or two, or more?

### How Separate Is MD from Other Disorders?

Two disorders that most frequently overlap diagnostically with depressive illness are anxiety and bipolar disorder. The prevailing view is that MD is highly comorbid with anxiety: about 60% of individuals with MD report a lifetime history of one or more anxiety disorders (Alonso et al., 2004a, Angst, 1993, Blazer et al., 1994, Hunt et al., 2004, Kessler et al., 1996, Kessler et al., 2003, Merikangas et al., 1996, Mineka et al., 1998, Pini et al., 1997, Zimmerman et al., 2008). The most closely related condition, symptomatically, is generalized anxiety disorder (GAD). Longitudinal studies indicate that while GAD precedes the occurrence of MD in about one-third of cases, conversely in about a third of cases, MD precedes GAD (Moffitt et al., 2007).

While there is general agreement in the literature for comorbidity between anxiety and MD, bipolar disorder and MD are usually thought to be separable. A distinction between unipolar (MD only) and bipolar (episodes of MD and mania) can be drawn on the basis that bipolar disorder’s onset age is on average 15 years younger than unipolar, recurs more frequently, is associated with different personality types (MD is associated with neuroticism and bipolar with sensation seeking or extraversion) (Perris, 1966b), and has an increased risk of bipolar illness in relatives (Gershon et al., 1982, Lieb et al., 2002, Weissman et al., 1984).

Genetics provides a way of testing the diagnostic uniqueness or otherwise of MD by determining the degree of genetic correlation between diseases. Do the same genetic loci that increase susceptibility to MD also increase susceptibility to other disorders? Two quantitative Reviews (meta-analyses) agree that there is a high genetic correlation between anxiety and MD (Cerdá et al., 2010, Middeldorp et al., 2005). Of 16 twin studies that report genetic covariation between anxiety and MD, all found that the genetic correlation between GAD and MD is not significantly different from unity. Demirkan and colleagues have recently confirmed the genetic correlation between MD and anxiety using SNP data to generate genetic risk scores (Demirkan et al., 2011). Thus, for anxiety, the comorbidity can be attributed, in part, to a common genetic basis. At a genetic level, GAD and MD are the same.

For many years, genetic data have been employed to support a separation of unipolar from bipolar affective illnesses: relatives of those with bipolar are more likely to develop bipolar, and conversely relatives of unipolar probands more likely to develop unipolar illness (MD, in other words) (Perris, 1966a). With few exceptions, subsequent studies have confirmed this observation: bipolar illness aggregates in the families of bipolar probands much more than in families of unipolar probands (Weissman et al., 1984). However, it is also true that in comparison to the general population, relatives of both bipolar and unipolar probands have increased risks of both forms of affective disorder (Gershon et al., 1982, Lieb et al., 2002, Weissman et al., 1984). The risk for bipolar disorder in relatives of MD probands is only modestly increased, approximately 2-fold across studies (on a relative risk scale) (Tsuang and Faraone, 1990). Conversely, there is about a 3-fold increase in risk of developing unipolar depression for a first-degree relative with bipolar disorder. Note that the base rates of unipolar and bipolar illnesses are very different: about 1% for bipolar as against 10% for unipolar. Altogether, a third to over a half of the affectively ill family members of bipolar patients manifest depressive illness (Weissman et al., 1984). Gershon argued from a study of 1,254 relatives of probands and controls that different affective disorders represent “thresholds on a continuum of underlying multifactorial vulnerability” (Gershon et al., 1982). If true, then bipolar disorder would be a more severe form of unipolar depression.

Genetic correlation data to test this hypothesis are limited: one twin study of 67 pairs of twins with bipolar and 177 with unipolar depression yielded a genetic correlation of 0.65 between the two disorders. However, the data were not consistent with the threshold model, namely that bipolar is a more severe subform of unipolar (McGuffin et al., 2003). A larger study of 486 twin pairs with affective illness provided some support for the threshold model, but the number of bipolar probands was small, so power to discriminate models was low (Kendler et al., 1995b).

Using SNP heritability approaches (So et al., 2011, Yang et al., 2011), there are now estimates of the genetic correlations between MD and bipolar disorder (Lee et al., 2013). The genetic correlation with bipolar disorder was 0.47 (SE 0.06), compatible with the twin-study genetic correlation of 0.64 (McGuffin et al., 2003). This finding suggests an overlap between unipolar and bipolar illnesses in which some loci contribute to both conditions. Consistent with this, genetic analysis of loci that act across disorders has been used to implicate calcium-channel signaling in the etiology of affective disorders (Cross-Disorder Group of the Psychiatric Genomics Consortium, 2013).

However, before concluding that molecular genetic analysis trumps the phenotypic separation of unipolar from bipolar, two points should be born in mind. GWAS results show that the majority of heritability can be assigned to many loci of small effects. How many that might be depends on the unknown contribution of rarer variants of large effect, but we can provide a rough estimate by assuming that depression is a quantitative trait, in which MD is one extreme (following the same reasoning for the power estimates for a successful MD GWAS [Yang et al., 2010b]). From the distribution of effect sizes of other quantitative traits, we can estimate the number of loci required to explain the heritability of MD. Assuming an exponential distribution (Goldstein, 2009), about 2,500 loci are required to explain half the heritability. This estimate is conservative, since the distribution of variants more closely follows a Weibull distribution than an exponential (Park et al., 2010). In short, the number of variants required to explain MD heritability implies that about one in five genes expressed in the brain are likely to be involved.

If thousands of variants confer susceptibility to MD, then this could explain a genetic correlation with other psychiatric disorders. We have no reason to expect the genetic architecture of anxiety, BP, or schizophrenia to be very different from MD: they are all likely to involve many loci of small effect, and they are all, at some level, brain disorders. Indeed, Ripke and colleagues estimate that 8,300 independent SNPs contribute to the genetic basis of schizophrenia, accounting for 50% of the variance in liability to schizophrenia (Ripke et al., 2013a). With 18,000 genes expressed in the brain (Lein et al., 2007), and each disorder influenced by variants in thousands of genes, genetic correlation may be inevitable.

The second point to note about the correlation between MD and other disorders concerns how well the phenotypic distinctions have been drawn. For example, no one has been able to identify features that distinguish with high accuracy episodes of MD in unipolar cases from episodes of MD in cases with bipolar illness. Furthermore, there is evidence that MD and BP share more characteristics than is sometimes appreciated: several authors have claimed that a large number of patients diagnosed with unipolar disorder have features of bipolar illness (Angst et al., 2010, Angst et al., 2011, Cassano et al., 2004, Zimmermann et al., 2009). When symptoms of subthreshold mania are sought (elevated mood, irritable mood, or increased activity), a large proportion of unipolar cases are found to qualify: up to half of all cases with unipolar illness (Angst et al., 2010, Angst et al., 2011, Zimmermann et al., 2009). However, subthreshold diagnoses depend critically on the quality of the assessments and the exact interpretation of what constitutes subclinical mania (it is easy to confuse a state of hypomania with elation from “normal” causes like falling in love, or getting a grant funded in grim times, or hyperactivity from the agitation that occurs in some depressive subtypes).

We can conclude that genetic and phenotypic classifications concur in identifying considerable overlap between anxiety and MD, with mixed support for a distinction between MD and bipolar disorder. The genetic data point to genetic overlap, but this may be, to some extent, a consequence of the polygenicity of complex traits. We turn next to the question of whether there exists a pure MD, rarer and harder to distinguish from bipolar than currently acknowledged, which has at least partly distinct genetic roots. Or more generally, we ask, are there genetically homogenous subtypes of MD?

### Is Major Depression One Disorder?

Those unfamiliar with the literature debating the division of MD into subtypes may be surprised not only at the diversity of the proposed classificatory systems employed (e.g., dimensional, hierarchical, or categorical) but also at the vehemence with which each position has been defended, or more usually attacked (Eysenck, 1970, Parker, 2000). The importance of this acrimonious debate is the extent to which genetic research strategies might resolve it and potentially guide interpretation of the underlying pathogenic mechanisms. Genetic data do in fact indicate heterogeneity. Most striking is the effect of sex.

As reviewed above, genetic effects on MD differ between men and women. It is more heritable in women and the genetic correlation between the sexes is approximately +0.60. To put this in perspective, the figure is comparable to the genetic correlations estimated between bipolar disorder and MD from twin studies (0.64; McGuffin et al., 2003) and SNP heritability (0.47; Lee et al., 2013). How can MD be one condition, when the degree of genetic correlation between the sexes is of the same magnitude as that between two supposedly separate disorders?

Heterogeneity is also evident at a phenotypic level. Currently, MD is diagnosed when depressed mood, or a loss of interest or pleasure in daily activities, is present for more than 2 weeks, and five or more out of nine symptoms (including low mood and loss of interest) occur nearly every day. Do these nine *DSM* symptomatic criteria for MD reflect a single underlying genetic factor? Surprisingly, only one study has addressed this question (Kendler et al., 2013). The best-fitting model to explain MD concordance in 7,500 adult twin pairs required three genetic factors, reflecting the psychomotor/cognitive, mood, and neurovegetative features of MD. As might have been predicted from a set of criteria chosen on the basis of clinical judgment rather than psychometric properties or validation from biological features, the nine *DSM* symptomatic criteria for MD do not appear to represent a single underlying genetic factor.

Second, do certain forms of MD breed true? That is to say, if we look in families, do we find that related individuals share similar phenotypic features? For example, some subjects report an atypical pattern of increased sleep and appetite (rather than the opposite); does this represent a heritable feature that might identify a genetically homogenous subtype? While some studies examining the inheritance of clinical features find that they do not breed true (Weissman et al., 1986, Weissman et al., 2006), latent class analysis of 14 symptoms of depression, assessed in 1,029 female twin pairs, revealed that members of a twin pair concordant for depression were significantly more likely than expected to share features of the latent class-derived syndrome (Kendler et al., 1996). This raises a third question, as to which, if any, features characterize an inherited form of MD.

Is there a more genetic form of MD? An old distinction between “endogenous” and “reactive” MD (Gillespie, 1929) is based upon the presumed occurrence of depressive episodes that were independent of precipitating events, compared to episodes that were an exaggerated reaction to life events. Is it possible that the endogenous form of MD is more genetically determined than others? The short answer to that question is no; in fact, contrary to the hypothesis that subjects whose MD appears to be devoid of precipitating events should have increased genetic predisposition (indexed by greater family history), the opposite is true: those reporting more stressful life events are more likely to have a family history (Kendler and Karkowski-Shuman, 1997). However, this finding does indicate, as the large literature on familial MD confirms (reviewed in Rutter et al., 1999, Sullivan et al., 2000), that clinical differences exist between those with and those without a family history of MD. Distinguishing features are relatively nonspecific: those with a family history of MD have more clinically severe illness, tend to present at an earlier age, and suffer higher rates of recurrence (Kendler et al., 1994, Kendler et al., 1999, Lieb et al., 2002, Weissman et al., 2006).

Environmental influences are also likely to stratify MD. Evidence from twin studies (Kendler et al., 1995a, Kendler et al., 2004, Silberg et al., 2001) indicate that genetic risk factors for MD not only alter average risk but also impact on sensitivity to the depressogenic effects of environmental adversities, particularly various forms of childhood maltreatment and recent stressful life events. The finding of increased genetic susceptibility to environmental stressors, or in short a gene by environment interaction, suggests the possibility of subdividing MD on the basis of environmental effects: theoretically genetic effects will be more homogeneous, relatively larger, and easier to detect in populations with clearly defined exposures.

While twin studies have shown that aggregate genetic risk factors for MD interact with stressful events, in recent years the field has been preoccupied with one of the many possible ways in which this effect might be explained at the molecular level. The dispute is whether or not the serotonin transporter *5-HTTLPR* variant is involved in a gene by environment interaction. The original study was carried out on a longitudinal cohort in New Zealand, and empirical literature dealing with whether that finding is robust, and replicable, is unclear and considerably polarized (Caspi et al., 2010, Kaufman et al., 2010, McGuffin et al., 2011).

Two meta-analyses found no evidence for an interaction (Munafò et al., 2009, Risch et al., 2009), while one meta-analysis concluded that there was an effect (Karg et al., 2011). The difference lies in the way studies were selected for the meta-analyses. The authors of the positive GxE meta-analysis take the view that the effect of GxE is broad: “rather than focus on a specific class of studies, we sought to perform a meta-analysis on the entire body of work assessing the relationship between *5-HTTLPR*, stress, and MD” (Karg et al., 2011). The one study that came closest to replicating the original design, a longitudinal study of a birth cohort in New Zealand, failed to replicate the first report (Fergusson et al., 2011). All that we can reasonably conclude is that current attempts to subdivide MD on the basis of interactions with environmental effects using candidate genes are unlikely to yield quick insights into the origins of the disease.

### Conclusion

Genetic analysis of MD was recently recognized to be among the greatest challenges facing health researchers (Collins et al., 2011). For some complex traits, including schizophrenia (Ripke et al., 2013a), there are now a number of verified genetic loci that contribute to disease susceptibility; in some cases, their discovery has implicated disease mechanisms, casting light on known, suspected, or indeed novel biological processes that explain why some people fall ill (Teslovich et al., 2010, van der Harst et al., 2012). Research findings in MD have yet to reach this stage. Despite convincing evidence for a genetic contribution to disease susceptibility, there has been a dearth of substantive molecular genetic findings. Nevertheless, there is an impressive quantity of relevant literature. Does it amount to anything? Yes, because negative findings impart important lessons.

The failure of GWAS analysis of more than 9,000 cases of MD (Ripke et al., 2013b) to find robust evidence for loci that exceed genome-wide significance is compatible with a paradigm in which the majority of the genetic variance is due to the joint effect of multiple loci of small effect. Twin studies and SNP-based heritability tests of the samples used for genome-wide association discount the possibility that there are no genetic effects to be found, leaving two nonmutually exclusive possibilities: either the effects are smaller than expected and/or the disorder is heterogeneous: different diseases might manifest with similar symptoms (incorrectly identified as the same illness), or there may be many different pathways to the same outcome (different environmental precipitants trigger MD in different ways, according to the genetic susceptibility of the individual).

We have reviewed evidence that indicates that MD is heterogeneous. This is clearly seen in the difference between sexes: genetics sees a greater difference between MD in men and MD in women than physicians recognize between anxiety and MD. However, while there is considerable agreement in the literature that MD has heterogeneous causes, there is much less agreement about its homogeneity as a clinical disease (Parker, 2000). Attempts to subdivide MD on the basis of inheritance have so far yielded only limited fruit: relatively nonspecific features, recurrence, and earlier onset indicate greater genetic predisposition.

The picture is consistent with a fairly undifferentiated phenotype emerging as the final common outcome of diverse processes, a process called equifinality in the development literature. The list of possible pathways is large: in addition to long-running favorites such as abnormalities of monoamine metabolism (including postreceptor components of the downstream cAMP signaling pathway [Duman et al., 1997]) and impaired corticosteroid receptor signaling (Holsboer, 2000), more recent hypotheses include the involvement of neurotrophins (Samuels and Hen, 2011), fibroblast growth factors (both ligands and receptors) (Turner et al., 2012), GABAergic deficits (Luscher et al., 2011), and epigenetic changes, specifically alterations in methylation and acetylation profiles at the promoters of glucocorticoid receptors and brain-derived neurotrophic factor (McGowan et al., 2009). Genetics does not support the primacy of one theory over another; indeed as our Review of the candidate gene literature indicates, genetics does not support any of the biological theories put forward to date.

### Recommendations

Our Review indicates two pathways forward. First, there is no reason to suppose that undifferentiated MD is intractable to GWAS, but success will require very large sample sizes (Figure 3). However, interpreting the results of such a study is likely to be challenging. We have seen that MD is highly comorbid with anxiety, and etiologically heterogeneous, at both genetic and environmental levels. Without information on comorbidity, known risk factors, and clinical phenotypes, the role of each locus will be unclear. Some will be sex specific, some will act only in situations of environmental stress, and others will predispose to anxiety. Genetic studies will need to include an extensive amount of phenotypic information if we are to make sense of hard-won mapping results.

Second, our Review indicates that we should not abandon attempts to concentrate on subtypes of MD. So far, studies using recurrent and early-onset MD have been no more successful than those that examine undifferentiated MD, but this may be due to lack of power. If we consider MD as part of a quantitative trait (representing liability to depression), then using a sample of more extreme cases would be equivalent to analyzing a rare disease (as Figure 3 demonstrates). Even a small improvement in genetic tractability could result in a large saving in the number of samples that need to be analyzed (reducing from 50,000 to 20,000, for example).

The problem is that we do not know for sure how to determine the scale on which severity is measured: is it the number of episodes of MD, the length of episodes, the number of symptoms, or some other feature or combination of features? Furthermore, the severity scale needs to differentiate cases with higher genetic risk, not those cases resulting largely from environmental adversities. Alternatively, subdividing MD on the basis of one or more clinical features (e.g., typical versus atypical vegetative features, standard versus postpartum onset), sensitivity to environmental stress, or sex, might identify a rarer, or at least a more genetically homogenous, subtype. At present, deciding which features to investigate is likely to be an ad hoc enterprise. Without knowing beforehand which to use, studies will need to be comprehensive, collecting as broad a range as possible of clinical features and known or putative risk factors.

Forty years ago, a perceptive Review of depressive disorders in *Science* (Akiskal and McKinney, 1973) argued that a psychoanalytic model of MD as object loss (a proximal cause of MD) could be conceptualized as loss of reinforcement, or loss of control over reinforcement, then subject to experimental investigation in animal models, and integrated with anatomical, biochemical, and pharmacological data as a process occurring in the diencephalic centers of reward. In this view, MD is a final common pathway, a decrease in the functional capacity of the reward system. Since then, MD has begun to appear as a relatively thin covering serving to unite a plethora of independently acting mechanisms. Genetic analyses can identify risk variants, both rare and common, and in so doing cast much needed illumination on the biology of the commonest psychiatric disorder. The difficulties of sample size and clinical differentiation are daunting but unavoidable if we are to take advantage of the promise that genetics makes.
