# The Genetics of Major Depression

**DOI:** 10.1016/j.neuron.2014.02.033

**Published:** 2014-03-05

**Authors:** Jonathan Flint, Kenneth S. Kendler

(Neuron *81*, 484–503; February 5, 2014)

The original publication contained errors in gene nomenclature in Table 2. The table has now been corrected in the article online.

